# Deletion of miR-150 Exacerbates Retinal Vascular Overgrowth in High-Fat-Diet Induced Diabetic Mice

**DOI:** 10.1371/journal.pone.0157543

**Published:** 2016-06-15

**Authors:** Liheng Shi, Andy Jeesu Kim, Richard Cheng-An Chang, Janet Ya-An Chang, Wei Ying, Michael L. Ko, Beiyan Zhou, Gladys Yi-Ping Ko

**Affiliations:** 1 Department of Veterinary Integrative Biosciences, College of Veterinary Medicine and Biomedical Sciences, Texas A&M University, College Station, Texas, United States of America; 2 Department of Veterinary Physiology and Pharmacology, College of Veterinary Medicine and Biomedical Sciences, Texas A&M University, College Station, Texas, United States of America; 3 Department of Immunology, University of Connecticut Health Center School of Medicine, Farmington, Connecticut, United States of America; 4 Texas A&M Institute of Neuroscience, Texas A&M University, College Station, Texas 77843–4458, United States of America; University of Illinois at Chicago, UNITED STATES

## Abstract

Diabetic retinopathy (DR) is the leading cause of blindness among American adults above 40 years old. The vascular complication in DR is a major cause of visual impairment, making finding therapeutic targets to block pathological angiogenesis a primary goal for developing DR treatments. MicroRNAs (miRs) have been proposed as diagnostic biomarkers and potential therapeutic targets for various ocular diseases including DR. In diabetic animals, the expression levels of several miRs, including miR-150, are altered. The expression of miR-150 is significantly suppressed in pathological neovascularization in mice with hyperoxia-induced retinopathy. The purpose of this study was to investigate the functional role of miR-150 in the development of retinal microvasculature complications in high-fat-diet (HFD) induced type 2 diabetic mice. Wild type (WT) and miR-150 null mutant (miR-150^-/-^) male mice were given a HFD (59% fat calories) or normal chow diet. Chronic HFD caused a decrease of serum miR-150 in WT mice. Mice on HFD for 7 months (both WT and miR-150^-/-^) had significant decreases in retinal light responses measured by electroretinograms (ERGs). The retinal neovascularization in miR-150^-/-^-HFD mice was significantly higher compared to their age matched WT-HFD mice, which indicates that miR-150 null mutation exacerbates chronic HFD-induced neovascularization in the retina. Overexpression of miR-150 in cultured endothelial cells caused a significant reduction of vascular endothelial growth factor receptor 2 (VEGFR2) protein levels. Hence, deletion of miR-150 significantly increased the retinal pathological angiogenesis in HFD induced type 2 diabetic mice, which was in part through VEGFR2.

## Introduction

Obesity associated type 2 diabetes has reached epidemic proportions in the past few decades (http://win.niddk.nih.gov/statistics/index.htm; provided in the public domain by the National Institute of Diabetes and Digestive and Kidney Diseases). Fluctuations in systemic glucose levels lead to serious complications such as diabetic retinopathy (DR), diabetic nephropathy, and diabetic neuropathy [1–3]. In diabetic retinopathy, microvascular complications often lead to leakage of blood into the eyes and ultimately cause retinal degeneration and distorted vision. Besides controlling systemic glycemic levels, intra-ocular anti-vascular endothelial growth factor (VEGF) injections are now the first-line gold standard treatment for DR macular edema and vascular complications [4,5]. While 70% of DR patients respond well to the current anti-VEGF therapies, about 30% are unresponsive [6–13], and often these non-responders are identified months after the treatments have started. In addition, side-effects such as sustained elevation of intra-ocular pressure, retinal detachments and tears, and ocular hemorrhage [14,15] become greater risks of blindness and can outweigh the benefits of anti-VEGF treatments. Hence, identifying the mechanistic development and discovering new strategies for treatment of DR are critical needs.

MicroRNAs are short non-coding RNAs that form complexes with RNA binding proteins to suppress the expression of targeted genes via post-transcriptional mechanisms [16]. MicroRNAs exhibit specific temporal and spatial expression following environmental stimulation [17], and they repress gene expression through complementary binding to the 3'UTR region of mRNAs. Because the expression of miRNAs can be cell or tissue specific, miRNAs have been used as diagnostic biomarkers or potential therapeutic targets [18–20]. In the retina, a single point mutation of miR-204 causes inherited retinal dystrophy [21]. In streptozotocin-induced diabetic rats, the expression levels of 86 miRNAs are altered in the retina, implying that these miRNAs might be involved in the pathogenesis of DR, and they could also serve as novel therapeutic targets for DR treatment [22–26].

Among these miRNAs that are altered in the diabetic retina, miR-150 is highly expressed in retinal vascular endothelial cells [27]. MiR-150 was first known as a circulating miRNA that is secreted into macrovesicles by blood cells and monocytic cells [28–30]. It regulates the development of B cells and production of hematopoietic progenitor cells [28–30]. MiR-150 expression or secretion is up-regulated by oxidative stress and down-regulated in hypoxic conditions [31,32] suggesting that it could be a biomarker coordinating cell to cell communication in the vascular system. The expression of miR-150 is significantly suppressed in pathological neovascularization in mice with hyperoxia-induced proliferative retinopathy [33]. This observation suggests that miR-150 is involved in neovascular complications in ocular diseases. Hence, we postulate that miR-150 might contribute to the pathogenesis of DR vascular complications.

We previously showed that obesity-induced early diabetes has a detrimental impact on retinal light sensitivity and health [34]. Mice under chronic high-fat-diet (HFD) not only become obese, they also develop a stable hyperglycemia with a progressively increased hyperinsulinemia over time, indicating the progressive worsening of insulin resistance [35]. These obese mice frequently develop chronic inflammation within adipose tissues [36–38], which is a significant factor contributing to systemic insulin resistance [38–42] and hyperglycemia [35], two hallmarks of type 2 diabetes. Unlike other diabetic mouse models derived from monogenic disorders or chemical destruction of β-cells, the HFD-induced diabetic mouse model is widely used in studies on pathophysiology of obesity and type 2 diabetes, such as mechanisms of impaired glucose tolerance and insulin resistance, as well as other diabetic complications [35,43], and it is more clinically relevant to obesity-associated type 2 diabetes in humans [35,43]. These HFD-induced diabetic mice also have structural and functional deficits in the retina similar to certain characteristics found in other DR animal models, including defects in the inner retinal light responses, lesions in the retinal vasculature, and thickness of Bruch's membrane [44]. Furthermore, mice fed with a HFD containing 42% fat calories for 12 months have significantly greater numbers of atrophic capillaries and pericyte ghosts compared to mice fed with a normal diet [45]. Because HFD-induced DR in mice is characterized by its slow-onset that mirrors the pathophysiology of human type 2 diabetes-associated DR, the HFD-induced DR mouse model is a suitable model for type 2 diabetes-associated non-proliferative DR in humans [45].

As shown in our previous study, mice under a HFD (59% fat calories) for 3 months not only become obese with insulin resistance and systemic hyperglycemia, their retinal light sensitivities decrease [34]. In this report, we extended our study and examined HFD-induced DR in mice after they were fed with a HFD for 7 months. We further examined the HFD-induced DR in miR-150 null mutant (miR-150^-/-^) mice and determined the functional role of miR-150 in neovascularization under diabetic conditions, as well as the downstream target(s) of miR-150 by using ERG recordings for retinal light responses, morphological staining for retinal vasculature, and other biochemical / molecular assays.

## Materials and Methods

### Animals

The C57BL/6J mouse strain was used in this study for its robust development of severe obesity, hyperglycemia, and hyperinsulinemia under the HFD regimen compared to other mouse strains [46]. Male C57BL/6J mice (wild type; WT) were purchased from Harlan (Houston, TX, USA). B6(C).MiR-150tm1Rsky/J (miR-150^-/-^) mice were originally purchased from the Jackson Laboratory (Bar Harbor, ME, USA). All animal experiments were approved by the Institutional Animal Care and Use Committee of Texas A&M University. Mice were housed under temperature and humidity-controlled conditions with 12:12 hour light–dark cycles. All mice were fed with laboratory chow and water *ad libitum*. Starting at 5 weeks of age, mice were fed either a HFD (59.4% fat calories, 18.1% protein calories, and 22.5% carbohydrates calories; TestDiet®, St. Louis, MO, USA) or normal standard laboratory chow (controls; 10% fat calories, 20% protein calories, and 70% carbohydrates calories; Research Diets, Inc., New Brunswick, NJ, USA) for 27 weeks. Their body weight and food intake were monitored throughout. Blood was taken from the tail vein and glucose levels measured by the Clarity Plus Blood Glucose Monitoring System (Diagnostic Test Group, Boca Raton, FL, USA).

### Electroretinogram (ERG)

Mice were dark adapted for a minimum of 6 hours and anesthetized with an intraperitoneal injection of Avertin (2% 2,2,2-tribromoethanol, 1.25% tert-amyl alcohol; Thermo Fisher Scientific, Grand Island, NY, USA) solution (12.5 mg/ml) at a dosage of 500 μl per 25 g bodyweight. Mice were placed on a heating pad to maintain body temperatures at 37°C. The ground electrode was placed on the tail, the reference electrode was placed under the skin in the cheek area below the eye, and the threaded recording electrode conjugated with a mini contact lens (OcuScience, Henderson, NV, USA) was placed on the surface of the cornea. A drop of Goniovisc (Hub Pharmaceuticals, Rancho Cucamonga, CA, USA) was applied on the surface of the cornea to keep it moist and to maintain proper contact between the cornea and the recording electrode. A portable ERG device (OcuScience) was used for the ERG recordings. The scotopic ERG recordings were carried out sequentially at light intensities of 0.1, 0.3, 1.0, 3.0, 10, and 25 cds/m^2^. A 1-minute recovery period was allowed between different intensities. Responses to four light flashes were averaged for the final ERG measurement at the lower light intensities (0.1, 0.3, 1.0, and 3.0 cds/m^2^), while only one light flash was applied for the higher light intensities (10 and 25 cds/m^2^). For the photopic ERG recordings, mice were first adapted to the background light at 30 cds/m^2^ for 10 minutes followed by exposure to a series of light stimulations from 0.1 to 25 cds/m^2^ with 32 flashes (0.5-second interval) at each light intensity. Responses to 32 light flashes were averaged for the final ERG measurement. The amplitudes and implicit time of a- and b-waves and oscillatory potentials (OPs) were recorded and analyzed by using the ERGView 4.4 software (OcuScience).

### Cell Culture and Western Immunoblot Analysis

Human umbilical vein endothelial cells (HUVECs, Cell Applications, San Diego, CA, USA) were seeded onto 24-well plates in endothelial cell growth medium (Cell Applications) and allowed to adhere overnight. The cells were transfected with a miR-150 expression vector (GeneCopoeia, Rockville, MD, USA) using an *in vitro* transfection kit (Signagen Lab, Rockville, MD, USA) and harvested 60 hours later. Human retinal endothelial cells (HRECs; Cell Systems, Kirkland, WA, USA) were maintained in EGM™-2MV BulletKit™ culture medium (Lonza, Allendale, NJ, USA). The cells were transfected with a miR-150 expression vector (GeneCopoeia) using the Lipofectamine LTX & PLUS reagent (Thermo Fisher Scientific) and harvested 60 hours after transfection. The cells were homogenized in RIPA buffer supplemented with a protease inhibitor mixture containing 1mM NaF and 1mM NaVO_3_. The cell lysates were centrifuged, and the supernatants were denatured with 2X SDS sample buffer. Samples were separated on 10% sodium dodecyl sulfate–polyacrylamide gels by electrophoresis and transferred to nitrocellulose membranes. The primary antibodies used in this study were anti-VEGFR2 (Cell Signaling Technology, Denver, MA, USA), anti-pan actin (Cell Signaling Technology), and anti-GAPDH (Cell Signaling Technology). Blots were visualized by using appropriate secondary antibodies conjugated to horseradish peroxidase (Cell Signaling Technology) and an enhanced chemiluminescence detection system (Thermo Fisher Scientific). Relative protein expressions for all proteins involved in this study were reported as a ratio to pan-actin. Band intensities were quantified by densitometry using Scion Image (National Institutes of Health, Bethesda, MD, USA). Each experiment was repeated four times (n = 4).

### Trypsin Digestion of Mouse Retinas and Hematoxylin and Eosin (H&E) Staining

Mice were deeply anesthetized by isoflurane and quickly decapitated to minimize pain and distress. Mouse eyes were excised and fixed with Zamboni's fixative (American Matertech Scientific Inc, Lodi, CA) for 2 hours at 4°C. The retinas were isolated and washed with distilled water overnight at room temperature. Trypsin digestion was performed to isolate the retinal vasculature according to a previous description [47]. Briefly, the retinas were incubated with 3% trypsin (BD Biosciences, Franklin Lakes, NJ, USA) for 1–1.5 h at 37°C, followed by gentle washing with distilled water until tissue debris no longer fell off the retinas. The remaining retinal tissues containing retinal blood vessels were carefully transferred to a glass-slide and flattened. The retinal vasculature was stained with heamatoxylin and eosin (H&E) using a staining kit (VitroView H&E Stain Kit, GeneCopoeia) or immunostained with the anti-VEGFR2 antibody conjugated with Alexa-488 (Cell Signaling) at 1:100 dilution.

### Immunofluorescent Staining and Quantification of Retinal Vasculature

For the analyses of retinal blood vessels, mouse eyes were dissected out at the end of the 27 week feeding regimen and fixed in Zamboni's fixative (VWR) for 2 hours at 4°C. The retinas were isolated and stained overnight at 25°C with FITC-conjugated Isolectin B4 (Sigma, St. Louis, MO, USA) in PBS containing 0.1% Triton X-100 and 1 mM Ca^2+^. Following 2 hours of washes, retinas were cut on the peripheral edge and flat-mounted with the photoreceptor side down onto microscope slides (VWR Scientific) in ProLong Antifade reagents (Thermo Fisher Scientific). Images were captured at 5x magnification on a Zeiss Digital Imaging Workstation (Zeiss, Thornwood, NY, USA), and whole retinal images were stitched together with the Image Composite Editor (Microsoft, Seattle, WA, USA). The vascular area (percentage, %) was quantified using Image J software (NIH, Bethesda, MD, USA) by the following procedure: in a selected retinal region, the total pixel-number of the selected area was first measured. Within the selected retinal region, the pixel-number of any green fluorescence-positive area was measured. The vascular area (%) was calculated by dividing the pixel-number of the fluorescence-positive area by the total pixel-number of the selected retinal region. There were 4 peripheral and 4 central areas from each whole retina randomly selected, analyzed, and averaged. The density of microaneurysm-like structures and the capillary vessel bifurcation joints (both together referred to as “microaneurysms”) were counted from 4 peripheral regions of each retina using the fluorescent images captured under 10X magnification. The number of microaneurysms was counted by Image J software. The microaneurysm particles (larger than 9 square pixels with circularity between 0.00–1.00) were highlighted and counted by adjusting the threshold. Each experimental group had retinas from 6–8 mice (n = 6–8).

### Quantitative Real Time PCR (Q-PCR)

Blood was collected from the mouse ocular vein and kept at room temperature for 30 minutes followed by centrifugation at 2000 g for 10 minutes at 4°C. 400–500 μl serum was transferred to a new tube and used for the purification of miRNAs (Direct-zol™ RNA Kit; Zymo Research, Irvine, CA, USA). Real time PCR reactions were performed by TaqMan miRNA assay kits (Thermo Fisher Scientific). Specific primers and probes for mmu-miR-150-5p (5’ UCUCCCAACCCUUGUACCAGUG 3’) were purchased from Life Technologies/Thermo Fisher. Taqman Q-PCR master mix (Thermo Fisher Scientific) was used for PCR amplification (40 cycles). All experiments were repeated five times (n = 5). A standard curve was generated with known quantities of miRNA (1 ×, 2 ×, 4 ×, 5 ×, 8 ×, and 10 × dilutions). The cycle values corresponding to the log values of the standard curve were used to generate an equation for linear regression. The cycle values from the sample miRNAs were fit into the equation to quantify miRNA levels. Cel-miR-39 (Life Technologies/Thermo Fisher) was used as the spike-in control.

### Statistics

All data are presented as mean ± standard error of the mean (SEM). Student’s *t*-test and one- or two-way ANOVA followed by Tukey's *post hoc* test for unbalanced n were used for statistical analyses. Specifically for the animal studies, the 2-way ANOVA followed by Tukey's *post hoc* test for unbalanced n were used to determine the statistical significance among all 4 groups (WT-normal diet, WT-HFD, miR-150^-/-^-normal diet, and miR-150^-/-^-HFD), as well as whether there was an interaction between the two factors: HFD regimen and miR-150 null mutation (miR-150^-/-^). A statistical significance in the interaction between HFD and miR-150^-/-^ would indicate that miR-150^-/-^ exacerbated the HFD-caused effects. Throughout, *p* < 0.05 was regarded as significant.

## Results

### Compared to the WT, deletion of miR-150 did not alter the systemic body weight and blood glucose level in HFD-induced obese mice

**Fig 1:**
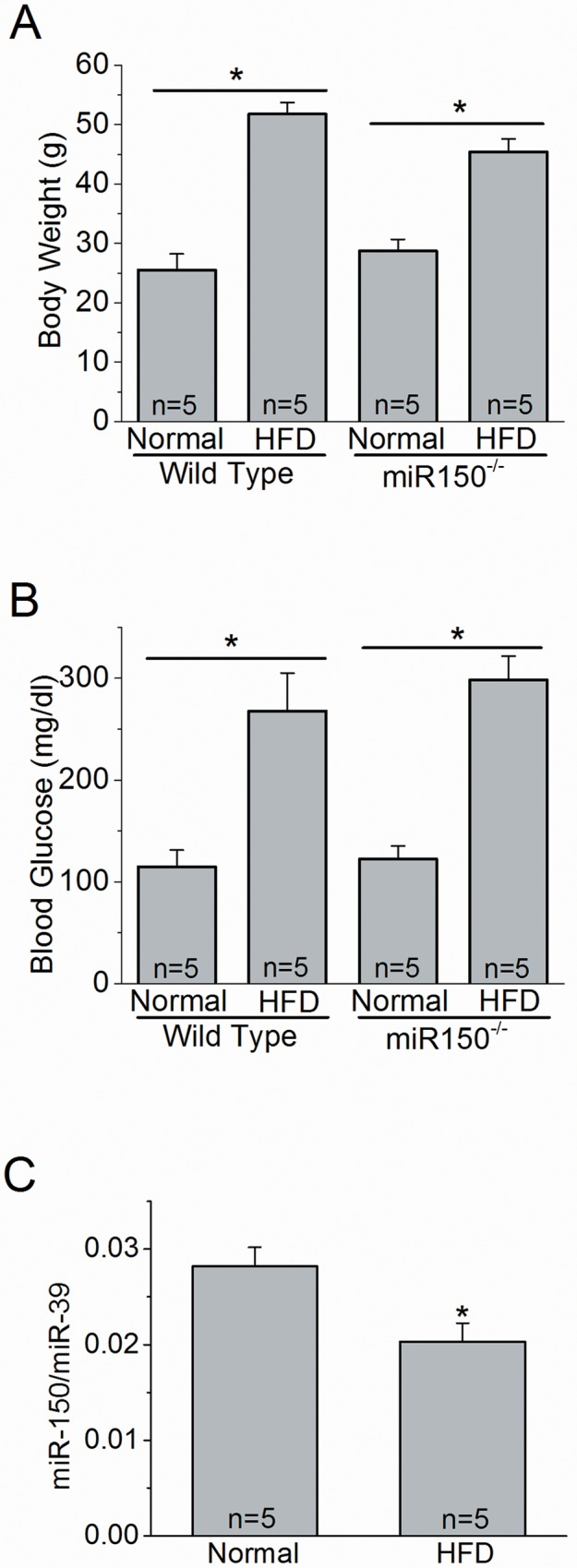
Systemic status of WT and miR-150 null mutant (miR-150^-/-^) mice with normal chow diet or HFD. Wild type (WT) and miR-150^-/-^ mice were fed with a normal chow diet (Normal) or HFD for 27 weeks. (A) The body weights and (B) blood glucose levels measured at the end of 27 weeks are presented. (A and B) There is a statistically significant difference between the HFD and Normal groups, but there is no statistical difference between WT-HFD and miR-150^-/-^-HFD. There is no statistical interaction between miR-150 null mutation and HFD regimen (2-way ANOVA). n = 5 for each group. (C) Wild type mice fed with HFD have a significant decrease of miR-150 expression compared to mice fed with normal diet (Normal; student’s *t*-test). *p < 0.05. miR-39 was used as the internal spike-control. The relative abundance of miR-150 / miR-39 for Normal is 0.02819 ± 0.00199, and for HFD is 0.02032 ± 0.00194. n = 5 for each group.

While overexpression of miR-150 in the mouse retina is able to block neovascularization in hyperoxia-induced retinopathy mice [33], it is unclear how miR-150 might affect retinal function under diabetic conditions. We examined both neuronal and vascular changes in the retina of age matched wild type (WT) and miR-150^-/-^ mice fed with a HFD for 27 weeks to determine the functional role of miR-150 in DR pathogenesis. We monitored the body weight and systemic blood glucose levels of WT and miR-150^-/-^ mice under normal chow and HFD conditions. By the end of the HFD regimen, both WT and miR-150^-/-^ HFD mice had twice the body weights of mice fed with a normal chow diet (WT: HFD vs normal chow, 51.8 ± 2.0 g vs 25.5 ± 2.7 g; miR-150^-/-^: HFD vs normal chow, 45.4 ± 2.2 g vs 28.7 ± 2.0 g; Fig 1A). There was no statistically significant difference in the interaction between miR-150 deletion and HFD regimen in regards to body weight, as the body weights between WT and miR-150^-/-^ mice in the same diet were similar (2-way ANOVA analysis). Both WT and miR-150^-/-^ HFD mice displayed significantly higher blood glucose levels in a non-fasted state compared to mice fed with normal chow, but there was no statistically significant difference in the interaction between miR-150 null mutation and HFD regimen in systemic glycemia, which indicated that hyperglycemia was mainly induced by chronic HFD (2-way ANOVA analysis; WT-normal diet: 114.8 ± 16.5 mg/dl; WT-HFD: 267.8 ± 37.5 mg/dl; miR-150^-/-^-normal diet: 122.4 ± 12.9 mg/dl; miR-150^-/-^-HFD: 298.2 ± 23.6 mg/dl; Fig 1B). Since miR-150 is largely present in the blood after it is expressed and secreted from the spleen, mesenteric lymph nodes, and the thymus [30], we next determined whether the serum level of miR-150 was altered by HFD. The serum level of miR-150 was significantly suppressed in HFD mice compared to mice fed with a normal diet (student’s *t*-test; the relative abundance of miR-150/ miR-39 for Normal is 0.02819 ± 0.00199, and for HFD is 0.02032 ± 0.00194. n = 5 for each group. Fig 1C). Therefore, miR-150 levels were influenced by diet, which could play a crucial role in the pathogenesis of DR.

### The retinal light responses were decreased in miR-150^-/-^ HFD mice

**Fig 2:**
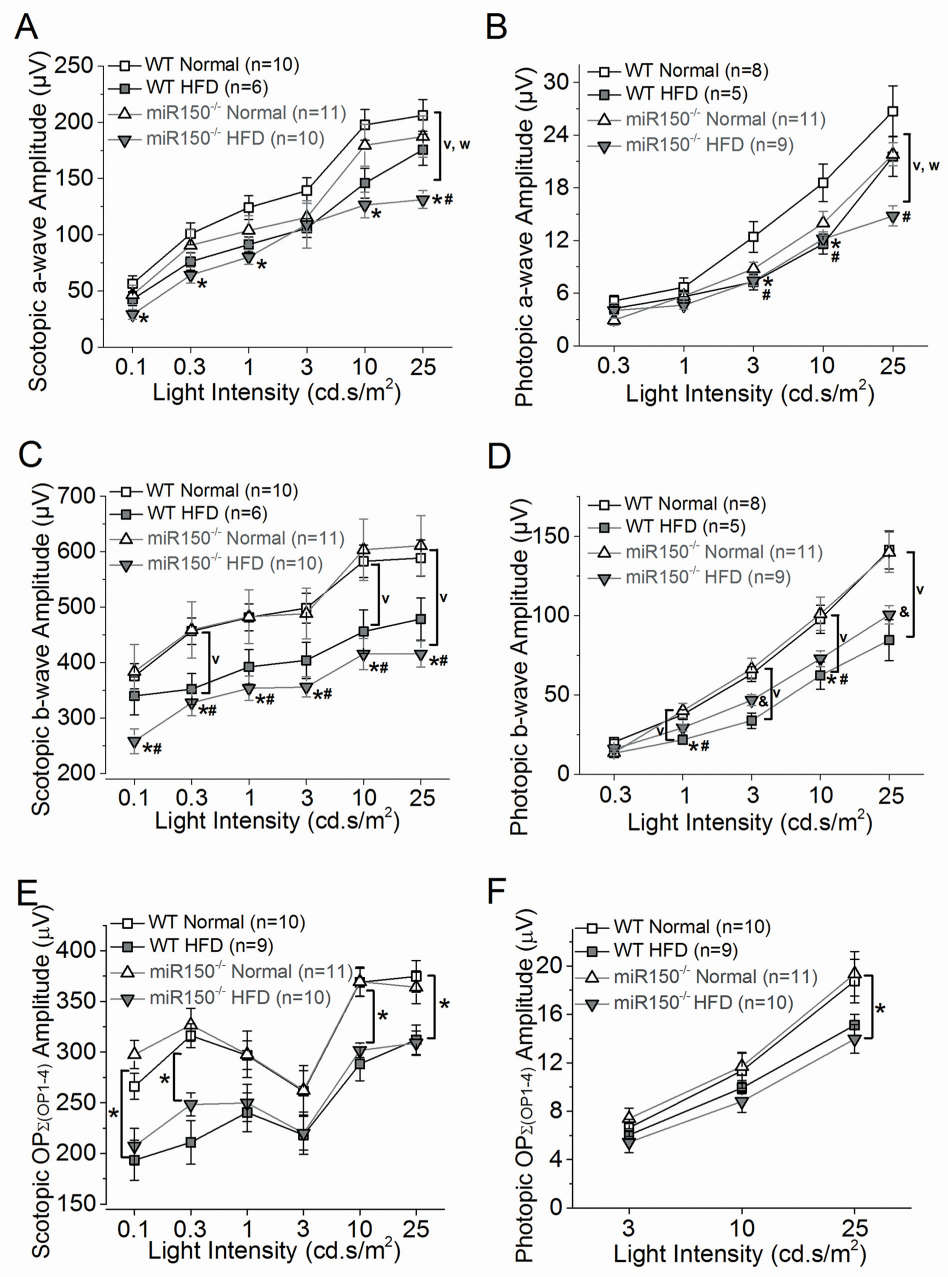
Scotopic and photopic light responses are decreased in WT and miR-150^-/-^ mice fed with HFD. All mice were dark adapted for at least 6 hours before ERG recordings. (A) The average scotopic ERG a-wave amplitudes recorded from miR-150^-/-^ with HFD (miR-150^-/-^-HFD) are significantly lower compared to the WT fed with normal chow diet (WT-Normal; *) or miR-150^-/-^ fed with normal chow diet (miR-150^-/-^-Normal; #). There is no statistical difference between WT fed with HFD (WT-HFD) and miR-150^-/-^-HFD. HFD-mice (both WT and miR-150^-/-^ groups) had significantly smaller (**v**) a-wave amplitudes compared to mice fed with a normal chow (both WT and miR-150^-/-^ groups). The miR-150^-/-^ mice (both normal chow and HFD groups) had significantly smaller (**w**) a-wave amplitudes compared to the WT mice (both normal chow and HFD groups). (B) The averaged photopic ERG a-wave amplitudes recorded from WT-HFD are significantly lower than WT-Normal (*) at 3 and 10 cd.s/m^2^ light intensities. The photopic a-wave amplitudes recorded from miR-150^-/-^-HFD are significantly lower than WT-normal (#) at 3, 10, and 25 cd.s/m^2^ light intensities. HFD-mice (both WT and miR-150^-/-^ groups) had significantly smaller (**v**) amplitudes compared to mice fed with a normal chow (both WT and miR-150^-/-^ groups). (C) The average scotopic ERG b-wave amplitudes recorded from miR-150^-/-^-HFD are significantly lower compared to WT-Normal (*) or miR-150^-/-^-Normal (#). There is no statistical difference between WT-HFD and miR-150^-/-^-HFD. HFD-mice (both WT and miR-150^-/-^ groups) had significantly smaller (**v**) a-wave amplitudes compared to mice fed with a normal chow (both WT and miR-150^-/-^ groups). The miR-150-/- mice (both normal chow and HFD groups) had significantly smaller (**w**) a-wave amplitudes compared to the WT mice (both normal chow and HFD groups). (D) The averaged ERG photopic b-wave amplitudes recorded from WT-HFD are significantly lower than WT-Normal (*) and miR-150^-/-^-Normal (#) at 1 and 10 cd.s/m^2^ light intensities. The photopic b-wave amplitudes recorded from miR-150^-/-^-HFD are significantly different from the other 3 groups (&) at 25 cd.s/m^2^ light intensities. HFD-mice (both WT and miR-150^-/-^ groups) had significantly smaller (**v**) amplitudes compared to mice fed with a normal chow (both WT and miR-150^-/-^ groups). (E) The averaged scotopic oscillatory potential amplitudes [as a summation from OP1 to OP4; Ʃ(OP1-4)] recorded from HFD-mice (both WT-HFD and miR-150^-/-^-HFD) are significantly lower (*) than mice fed with a normal chow (both WT-Normal and miR-150^-/-^-Normal) at 0.1, 0.3, 10, and 25 cd.s/m^2^ light intensities. (F) The averaged photopic oscillatory potential amplitudes [Ʃ(OP1-4)] recorded from HFD-mice (both WT-HFD and miR-150^-/-^-HFD) are significantly lower (*) than mice fed with a normal chow (both WT-Normal and miR-150^-/-^-Normal) at 25 cd.s/m^2^ light intensities. (A-F) Overall, there is no statistical significance of interaction between two factors: miR-150 null mutation and HFD regimen (2-way ANOVA). *p* < 0.05 (denoted as *, #, &, v, w). Data of scotopic and photopic ERG a- and b-waves and OPs are listed in Tables 1 and 2.

We further examined whether the retinal light responses were compromised in miR-150^-/-^ null mutant mice at the end of a HFD regimen for 27 weeks. Using electroretinogram (ERG) recordings, we measured the retinal light responses under scotopic or photopic conditions in the following 4 experimental groups: WT with normal chow diet (WT-Normal; n = 10), WT with HFD (WT-HFD; n = 6), miR-150^-/-^ with normal chow diet (miR-150^-/-^-Normal; n = 6), and miR-150^-/-^ with HFD (miR-150^-/-^-HFD; n = 10). ERG a- and b-waves and oscillatory potentials (OPs) were recorded and analyzed at various light intensities under scotopic or photopic conditions. The ERG a-wave represents the activities of hyperpolarization of photoreceptors upon light stimulation, and the b-wave represents the light-evoked inner retinal responses, which reflects the summation of responses from bipolar, Müller, and amacrine cells [48,49]. The OPs reflect the inner retinal responses with the currents largely generated from amacrine cells [49]. Severely reduced or abolished OPs are found in patients with advanced DR [50]. Mice were dark adapted for at least 6 hours before the scotopic ERG recordings. For the photopic ERG recordings, mice were first exposed to 30 cd.s/m^2^ background light for 10 minutes followed by exposure to various light-intensity stimulations. In general, the average ERG a- and b-wave amplitudes of mice with normal chow diet were higher than those of HFD-mice (Fig 2 and Tables 1 and 2), which echoed our previous finding that HFD mice have significant decreases of ERG a- and b-wave responses compared to mice fed with normal chow [34]. MiR-150^-/-^ mice (both the normal chow and HFD groups) had lower ERG a-wave amplitudes (scotopic and photopic) compared to the WT mice (both the normal chow and HFD groups), indicating that miR-150 deletion might negatively impact photoreceptor light responses (Fig 2A and 2B). However, there was no statistically significant difference in the interaction between the two factors, HFD regimen and miR-150 null mutation, on ERG a-waves (2-way ANOVA), even though miR-150^-/-^ mice fed with HFD had the lowest a- and b-wave amplitudes under both scotopic and photopic conditions compared to the other three experimental groups. In scotopic and photopic b-waves (Fig 2C and 2D), HFD-mice (both WT and miR-150^-/-^ groups) had significantly decreased amplitudes compared to mice fed with normal chow (both WT and miR-150^-/-^ groups). MiR-150 deletion did not have any impact on ERG b-wave amplitudes, suggesting that miR-150 deletion did not affect inner retinal physiology. In addition, there was no statistically significant difference in the interaction between the two factors, miR-150 null mutation and HFD regimen (2-way ANOVA). The implicit time of a- and b-waves under scotopic and photopic conditions showed similar statistical comparisons among all four groups (data not shown).

**Table 1:** Dark-adapted (scotopic) retinal light responses (Data for Fig 2A, 2B and 2E).

Light Intensity cd·s/m^2^	WT-Normal	WT-HFD	miR-150^-/-^-Normal	miR-150^-/-^-HFD
**Scotopic a-wave amplitude (μV)**				
**0.1**	56.5±7.0	42.6±5.6	46.6±8.5	29.1±4.4 *
**0.3**	100.8±9.7	76.1±7.8	90.6±14.0	64.1±7.0 *
**1**	124.2±10.5	91.3±6.7	103.8±13.0	80.2±6.6 *
**3**	139.3±11.4	105.8±8.3	115.5±12.6	109.2±20.9
**10**	197.7±13.6	145.9±13.2	179.6±18.7	126.4±11.4 *
**25**	206.2±14.1	175.6±13.9	187.3±18.5	131.2±7.9 *^,^ ^#^
**Scotopic b-wave amplitude (μV)**				
**0.1**	375.3±22.9	339.9±34.1	383.9±48.8	258.3±22.4 *^,^ ^#^
**0.3**	456.8±24.0	352.3±27.8	459.2±50.8	327.7±23.4 *^,^ ^#^
**1**	481.7±24.5	392.3±31.2	482.8±48.5	353.6±21.9 *^,^ ^#^
**3**	498.4±26.8	404.1±32.7	488.4±45.7	356.0±18.0 *^,^ ^#^
**10**	582.8±29.4	456.0±39.1	603.7±55.7	415.5±28.2 *^,^ ^#^
**25**	588.7±32.4	478.8±38.0	611.0±54.3	415.7±23.6 *^,^ ^#^
**Scotopic Oscillatory Potentials: ƩOP (1–4) amplitude (μV)**				
**0.1**	266.2±12.9	193.4±19.7^	197.6±13.8	207.2±17.6^
**0.3**	316.3±11.8	211.0±21.5^	326.9±16.3	248.3±11.4^
**1**	296.9±14.0	240.5±19.1	297.8±23.0	249.8±18.4
**3**	261.4±20.4	218.2±19.0	262.34±24.5	220.0±16.8
**10**	368.9±13.9	288.3±16.5^	369.5±14.3	301.7±7.1^
**25**	374.7±15.7	312.1±14.6^	364.0±16.24	308.9±12.0^

**Scotopic ERG a-wave:**

* denotes miR-150-/—HFD significantly different from WT-Normal.

# denotes miR-150-/—HFD significantly different from miR-150-/—Normal.

There is a significant difference between mice with normal chow (both WT and miR-150-/-) and HFD-mice (both WT and miR-150-/-), indicating the impact of HFD on the scotopic ERG a-wave.

There is a significant difference between WT (both normal chow and HFD) and miR-150-/-, indicating the impact of miR-150 null mutation on the scotopic ERG a-wave.

**Scotopic ERG b-wave:**

* denotes miR-150^-/-^-HFD significantly different from WT-Normal.

^**#**^ denotes miR-150^-/-^-HFD significantly different from miR-150^-/-^-Normal.

There is a significant difference between mice with normal chow (both WT and miR-150^-/-^) and HFD-mice (both WT and miR-150^-/-^), indicating the impact of HFD on the scotopic ERG b-wave.

**Scotopic ERG Oscillatory Potentials:**

^ denotes both WT-HFD and miR-150-/—HFD significantly different from both WT-Normal and miR-150-/—Normal, indicating the impact of HFD on scotopic ERG OPs.

**Table 2:** Light-adapted (photopic) retinal light responses (Data for Fig 2C, 2D and 2F).

Light Intensity cd·s/m^2^	WT-Normal	WT-HFD	miR-150^-/-^-Normal	miR-150^-/-^-HFD
**Photopic a-wave amplitude (μV)**				
**0.3**	5.1±0.6	4.3±0.6	2.9±0.6	4.0±0.8
**1**	6.7±1.1	5.6±0.7	5.7±0.3	4.6±0.4
**3**	12.4±1.7	7.3±0.9 *	8.8±0.8	7.4±0.5 ^#^
**10**	18.6±2.1	11.6±1.1 *	14.0±1.4	12.2±0.8 ^#^
**25**	26.7±2.9	21.6±2.3	21.8±1.3	14.8±1.2 ^#^
**Photopic b-wave amplitude (μV)**				
**0.3**	20.4±1.6	13.3±2.3	13.6±1.7	15.9±2.0
**1**	37.6±3.2	21.8±2.9 *^,^ ^#^	40.2±4.6	29.4±2.1
**3**	63.2±4.6	33.8±4.8	66.5±6.7	46.7±3.4 ^&^
**10**	97.8±8.9	62.3±8.6 *^,^ ^#^	101.2±10.5	72.9±5.0
**25**	141.5±12.0	84.6±13.0	140.0±12.7	100.6±5.8 ^&^
**Photopic Oscillatory Potentials: ƩOP (1–4) amplitude (μV)**				
**3**	6.63±0.69	6.03±0.37	7.40±0.86	5.44±0.87
**10**	11.3±1.4	9.92±0.47	11.7±1.2	8.81±0.90
**25**	18.8±1.8	15.1±0.9^	19.4±1.8	14.0±1.2^

**Photopic ERG a-wave:**

* denotes WT-HFD significantly different from WT-Normal.

# denotes miR-150-/—HFD significantly different from WT-Normal.

There is a significant difference between mice with normal chow (both WT and miR-150-/-) and HFD-mice (both WT and miR-150-/-), indicating the impact of HFD on the photopic ERG a-wave.

There is a significant difference between WT (both normal chow and HFD) and miR-150-/-, indicating the impact of miR-150 null mutation on the photopic ERG a-wave.

**Photopic ERG b-wave:**

* denotes WT-HFD significantly different from WT-Normal.

# denotes WT-HFD significantly different from miR-150-/—Normal.

& denotes miR-150-/—HFD significantly different from all other 3 groups.

There is a significant difference between mice with normal chow (both WT and miR-150-/-) and HFD-mice (both WT and miR-150-/-), indicating the impact of HFD on the photopic ERG b-wave.

**Photopic ERG Oscillatory Potentials:**

**^** denotes both WT-HFD and miR-150^-/-^-HFD significantly different from both WT-Normal and miR-150^-/-^-Normal, indicating the impact of HFD on photopic ERG OPs.

The amplitudes of OPs (the summation of OP1, OP2, OP3, and OP4; ƩOP1-4) were significantly decreased in HFD-mice (both WT and miR-150^-/-^) compared to the mice fed with normal chow (Fig 2E and 2F, Tables 1 and 2). However, because the photopic OP amplitudes were too small to be analyzed at lower light intensities, we were only able to obtain photopic OPs from 3, 10, and 25 cd.s/m^2^, while we were able to analyze scotopic OPs from all light intensities tested in this study (from 0.1 to 25 cd.s/m^2^). Deletion of miR-150 itself did not affect OPs. There was no statistically significant difference in the interaction between the two factors, miR-150 null mutation and HFD regimen (2-way ANOVA). These results indicated that HFD had a detrimental impact on the inner retina, and deletion of miR-150 did not further aggravate the decreased retinal light responses in HFD-induced diabetic mice.

### Deletion of miR-150 exacerbates HFD-induced DR microvascular complications

**Fig 3:**
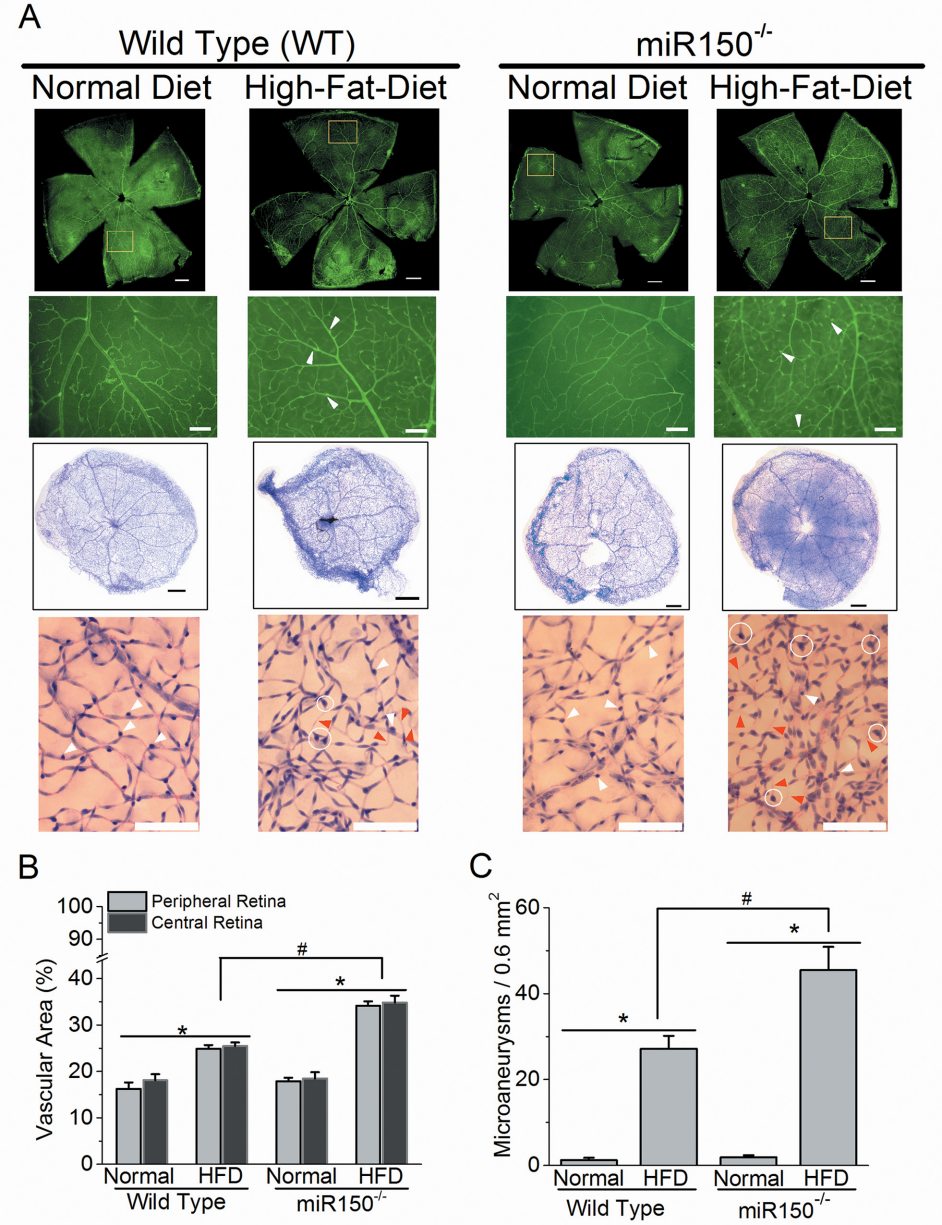
Deletion of miR-150 exacerbates HFD-induced DR neovascularization and microaneurysms. (A) Upper two rows: the whole mount retinal vasculature was stained with FITC-labeled isolectin-B4. The first row: the fluorescent images from 4 experimental groups were taken at 5X (scale bar = 400 μm). The highlighted regions (yellow square) were magnified at 10X and displayed in the second row. The second row: the fluorescent images from 4 experimental groups were taken at 10X (scale bar = 100 μm). Lower two rows: the mouse retinas were trypsin-digested and the retinal vasculatures were stained with hematoxylin and eosin. The third row: the whole retinal vasculature images are shown (scale bar = 400 μm). The fourth row: magnified images of retinal vasculature (from the third row) are shown (scale bar = 100 μm). White arrowheads indicate the pericytes, the red arrowheads indicate the acellular capillaries, and the white circles indicate the microaneurysm-like (vascular extrusion) structures. (B) Wild type and miR-150^-/-^ mice fed with HFD have significantly higher (*) vasculature in central and peripheral retinal areas compared to mice fed with normal chow diet (Normal). There is a statistical significant difference in the interaction between miR-150 null mutation and HFD regimen (2-way ANOVA; #). (C) Wild type and miR-150^-/-^ mice fed with HFD have significantly higher (*) densities of microaneurysm-like structures (microaneurysms per 0.6 mm^2^ retinal area) compared to mice fed with normal chow diet (Normal). There is a statistical significant difference in the interaction between miR-150 null mutation and HFD regimen (2-way ANOVA; #). WT-Normal: n = 6; WT-HFD: n = 8; miR-150^-/-^-Normal: n = 6; miR-150^-/-^-HFD: n = 8. *p* < 0.05 (denoted as *, #).

Even though miR-150 mutation did not exacerbate HFD-induced decreases of retinal light responses, we further examined whether deletion of miR-150 affected neo-microvasculature by fluorescent staining of retinal blood vessels. Mice fed with HFD for 27 weeks had a significant increase in retinal vascular densities measured in central and peripheral retinas (Fig 3A and 3B). To better observe the changes in retinal vasculature, we performed trypsin-digestion on mouse retinas to isolate the retinal vasculature. The retinal neurons and other nonvascular cells were washed out after trypsin digestion, and the retinal vasculature was visualized by H&E staining. Retinal capillary vasculature is composed of retinal endothelial cells and pericytes. The endothelial cells have an elongated shape (Fig 3A) and form the tube of capillaries, while pericytes with a rounded shape (white arrowheads, Fig 3A) wrap around the endothelial cells and sustain the blood–retina barrier. We observed several defects of retinal vasculature from the mice fed with HFD (Fig 3A), including the loss of pericytes and the formation of acellular capillaries (red arrowheads), both of which occur in early human DR. Thus, mice under HFD for 27 weeks had developed DR.

Deletion of miR-150 itself did not seem to cause any vascular abnormality in the mouse retina, but miR-150^-/-^ mice fed with HFD had significantly increased vasculature networks (Fig 3A). In addition to the pericyte loss and the formation of acellular capillaries, we also identified microaneurysm-like structures (endothelial cell-extrusion from existing capillaries) in the HFD-mouse retinal vasculature. As these microaneurysm-like structures (white circles; Fig 3A) were smaller compared to human DR microaneurysms, it is possible that these structures could be newly formed branch points (bifurcations), or they could develop into larger structures that resemble the human DR microaneurysms in a later stage of DR. However, we observed these bifurcations and microaneurysm-like structures (“microaneurysms”) mostly in HFD mouse retinal vasculature but rarely in mice fed with normal chow, and the density of these microaneurysms was significantly higher in HFD-mice compared to mice fed with normal chow (Fig 3C). Moreover, there was a statistically significant difference in the interaction between miR-150 mutation and HFD regimen in vascular area and microaneurysms (^#^ *p* <0.05; 2-way ANOVA; Fig 3B and 3C) indicating that miR-150^-/-^ mice are more sensitive to HFD-induced diabetic insults during the development of DR vascular complications compared to the WT. Thus, deletion of miR-150 accelerated the pathological neovascularization in HFD-induced diabetic retina.

### Vascular endothelial growth factor receptor 2 (VEGFR2) is a downstream target of miR-150

**Fig 4:**
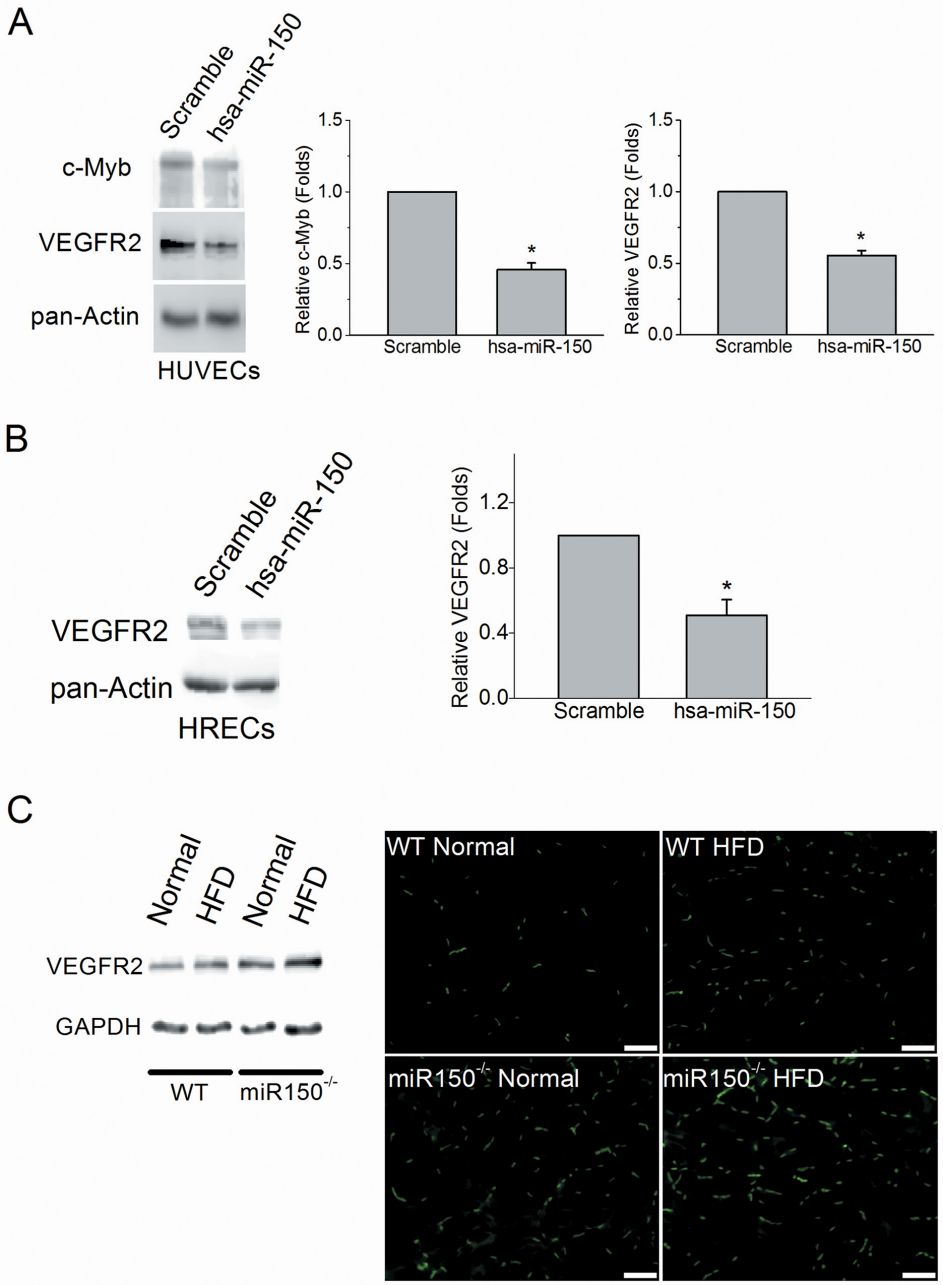
Overexpression of miR-150 decreased VEGFR2 protein level in endothelial cells. The HUVE cells were transfected with miR-150 (has-miR-150) or a scramble microRNA (Scramble) and cultured for an additional 60 hr. The protein levels of c-Myb and VEGFR2 are significantly lower in HUVE cells with overexpression of miR-150 compared to the scramble (student’s *t*-test; *p < 0.05). n = 4 for each group. (B) The protein level of VEGFR2 is significantly lower in HRECs transfected with has-miR-150 compared to the ones transfected with scramble (student’s t-test; *p < 0.05). n = 4 for each group. (C) The retinas from WT and miR-150^-/-^ mice under normal chow diet (Normal) or HFD were isolated and processed for Western blotting. Some retinas were trypsin-digested to obtain the retinal vasculature followed by immunostaining with the VEGFR2 antibody conjugated with Alexa-488. The scale bar = 100 μm.

Angiogenic factors including VEGF, fibroblast growth factor (FGF), platelet-derived growth factor (PDGF), and angiopoietins promote endothelial cell proliferation and vascular tube formation through their respective receptors [51]. Vascular endothelial growth factor receptor 2 (VEGFR2) is one of the principal receptors of VEGF, which mediates VEGF centric signaling [51]. Upregulation of VEGF has been observed in hypoxia-induced retinopathy, diabetic retinopathy, and age-dependent macular degeneration [52]. Since deletion of miR-150 increased microvascular density and angiogenesis in mouse retina under diabetic conditions, we suspected that miR-150 might regulate VEGF signaling. To determine whether miR-150 was able to regulate the VEGFR2 level in endothelial cells, we transfected human umbilical vein endothelial cells (HUVECs) with a miR-150 expression vector and detected the protein levels of VEGFR2. Myeloblastosis (c-Myb) is one of the known targets of miR-150 [10,53–55] and as such was used as a positive control. Overexpression of miR-150 (hsa-miR-150) in HUVECs caused a decrease of VEGFR2 (0.55 ± 0.03 fold) and c-Myb (0.46 ± 0.04 fold) compared to the control transfected with a scramble microRNA (Scramble, 1.00 ± 0.09 fold; Fig 4A). A similar result was observed in the transfected human retinal microvascular endothelial cells (HRECs). Overexpression of miR-150 in HRECs inhibited VEGFR2 level (0.51 ± 0.10 fold) compared to the control scramble (1.00 ± 0.21 fold; Fig 4B). In addition, we found that the VEGFR2 level in the miR-150^-/-^ mouse retinas was higher than the age matched WT mouse retinas (under both normal chow and HFD; Fig 4C). The presence of VEGFR2 in the retinal vasculature obtained from the miR-150^-/-^ under HFD was higher than that of the WT (Fig 4C). Thus, the ability of miR-150 to regulate angiogenesis is in part through VEGFR2.

## Discussion

Previously, we observed that obesity-induced type 2 diabetic conditions decrease retinal light sensitivities [34]. Mice fed with a HFD for only 3 months developed chronic insulin-resistance and glucose intolerance with significantly increased adiposity, which are signs of type 2 diabetes [34]. In this study, we found that the serum miR-150 level was significantly suppressed in WT HFD-induced type 2 diabetic mice (Fig 1). By using the miR-150 null (miR-150^-/-^) mouse model, we were able to reveal how miR-150 contributed to the pathogenesis of DR vascular complications in HFD-induced type 2 diabetes. Deletion of miR-150 in mice mildly affected photoreceptor light sensitivities (as shown by ERG a-waves), but it did not have any significant impact on the inner retinal light sensitivities (shown in ERG b-waves and OPs) or general retinal vasculature. However, under HFD-induced diabetic conditions, miR-150^-/-^ mice had a significant increase in retinal vasculature and microaneurysms compared to the WT-HFD mice (Fig 3), even though retinal light responses were similar. Thus, deletion of miR-150 does not further exacerbate the neural retina as much as ocular vasculature under diabetic insults. This observation might be due to the fact that normally the neural retina has very low expression of miR-150 compared to the hematologic and endothelial cells in the blood vessels [27,31], so under chronic diabetic stress, deletion of miR-150 has a much higher pathological impact on ocular vasculature than neural retina.

In DR, intra-ocular anti-VEGF injections and anti-inflammatory therapies are the standard treatments for DR macular edema and vascular complications [1,4,5]. Several microRNAs including circulating miRNAs have been reported to play a critical role in pathological angiogenesis by targeting angiogenic factors including VEGF, FGF, PDGF, or angiopoietins. For example, under hyperglycemic conditions, miR-93 and miR-200b regulate VEGF levels in the kidney and retina, respectively [56,57]. MiR-126, miR-31, miR-150, and miR-184 are involved in ischemia-induced retinal neovascularization. MiR-126 enhances the action of VEGF and FGF by repressing the expression of Spred-1, an intracellular inhibitor of angiogenic signaling [58]. Downregulation of miR-31, miR-150, and miR-184 in ischemic retina stimulates ocular neovascularization by increasing VEGF and PDGF [32]. Among these miRNAs mentioned above, miR-150 and miR-126 are circulating miRNAs present in the plasma, platelets, erythrocytes, and nucleated blood cells of blood [59,60]. Our data presented in this report show that miR-150, a circulating miRNA but also expressed in retinal endothelial cells [27], contributes to the pathogenesis of DR vascular complications. Our work broadens the knowledge of circulating miRNAs during DR pathogenesis.

In a mouse model of oxygen-induced proliferative retinopathy, miR-150 is a suppressor of pathological ocular neovascularization [33]. However, whether VEGF, the most potent angiogenic factor, is a direct target of miR-150 remains controversial [33,34,61–64]. Our results suggest that the suppression of angiogenesis by miR-150 is in part through VEGFR2, a principal receptor of VEGF that mediates specific intracellular signaling cascades leading to proliferation, migration, survival, and permeability of vascular endothelial cells [65]. One question that remained is whether VEGFR2 is a direct downstream target of miR-150. We performed a miRNA target prediction search using several on-line algorithms including Targetscan (http://www.targetscan.org), PicTar (http://pictar.mdc-berlin.de), and miRanda (http://www.microrna.org) to determine whether miR150 could target the 3’-untranslated region (UTR) of VEGFR2. However, the gene encoding *VEGFR2 (KDR)* is not listed as a potential candidate for miR-150 because of a lack of compatible paired sequences. Conversely, the miRNAs predicted to target the 3’-UTR (1479 bp) of mouse *VEGFR2* gene do not include miR-150. Therefore, we concluded that VEGFR2 is not a direct downstream target of miR-150. The down-regulation of VEGFR2 by miR-150 could be indirect and mediated by other known miR-150 targets, such as c-Myb, Early growth response 2 (Egr2), or Glycoprotein nonmetastatic melanoma protein B (GPNMB) [10,53–55]. Among those candidates targeted directly by miR-150, c-Myb has been verified as a consensus target in several studies [10,29,66]. Our result showed that the c-Myb level was decreased in the endothelial cells transfected with miR-150. C-Myb is a transcriptional factor that binds to a 5’-YAACKG-3’ sequence in the promoter region and regulates the expression of a group of genes involved in cell lineage and fate- determination in the immune system. The gene encoding VEGFR2 (*Vegfr2*) has four c-Myb binding sites in its promoter region, so *Vegfr2* can be turned on by c-Myb [67]. Over-expression of c-Myb increases the population of hemogenic endothelial cells during embryonic development [68]. Therefore, c-Myb is the most likely downstream target of miR-150 that regulates the VEGFR2 expression in endothelial cells.

However, there are other direct targets of miR-150 that could also contribute to the regulation of VEGFR2 expression. Egr2 induces VEGF and VEGFR2 expression in Schwann cells and possibly upregulates expression of both during embryonic development and angiogenesis [69,70]. Therefore, downregulation of Egr2 by miR-150 inhibits VEGFR2 activation. GPNMB is a glycoprotein with the ability to induce endothelial cell migration [71]. MiR-150^-/-^ null neonates display an increased capillary network, decreased inflammation, and less alveolar damage after hyperoxia-induced lung injury due to concurrent induction of GPNMB expression [55]. GPNMB increases the expression of neuropilin-1 (NRP-1) that forms a complex with VEGFR2 to enhance tumor cell-intrinsic VEGF signaling and primary breast tumor growth [72]. All of the above support the notion that even though VEGFR2 might not be a direct target of miR-150, deletion of miR-150 enhances angiogenesis under pathological conditions in part through the up-regulation of VEGFR2.

In addition to anti-VEGF therapies, anti-inflammatory treatments have also been used to treat DR [1,4,5]. The miR-150^-/-^ mice display exacerbated obesity-associated tissue inflammation and systemic insulin resistance [73], indicating that miR-150 might be involved in the chronic ocular tissue inflammation that leads to the development of DR. We previously showed that mice fed with a HFD for only 3 months display an increase of inflammation in the retina [34]. Since HFD-induced diabetes exacerbated the DR neovascularization in miR-150^-/-^ mice more than in WT (Fig 3), and overexpression of miR-150 significantly down-regulated VEGFR2 in endothelial cells (Fig 4), we postulate that chronic obesity-induced suppression of miR-150 may lead to the inflammation in ocular tissues and further aggravate the DR neovascularization through up-regulating VEGF/VEGFR2. Because the pathogenesis of DR neovascularization is complex, and the development of DR vascular complication is chronic, it is our interest to investigate the role of miR-150 in pathological angiogenesis at different stages of DR development. Nonetheless, miR-150 might be a potential new therapeutic target to reduce the retinal vascular complications caused by DR or other retinal degenerative diseases because of its functional association with inflammation and neovascularization.
